# X-ray induced dimerization of cinnamic acid: Time-resolved inelastic X-ray scattering study

**DOI:** 10.1038/srep15851

**Published:** 2015-11-16

**Authors:** Juho Inkinen, Johannes Niskanen, Tuomas Talka, Christoph J. Sahle, Harald Müller, Leonid Khriachtchev, Javad Hashemi, Ali Akbari, Mikko Hakala, Simo Huotari

**Affiliations:** 1 Department of Physics, P.O. Box 64, FI-00014 University of Helsinki, Helsinki, Finland; 2 ESRF - The European Synchrotron, CS 40220, F-38043 Grenoble Cedex 9, France; 3 Department of Chemistry, P.O. Box 55, FI-00014 University of Helsinki, Helsinki, Finland

## Abstract

A classic example of solid-state topochemical reactions is the ultraviolet-light induced photodimerization of α-trans-cinnamic acid (CA). Here, we report the first observation of an X-ray-induced dimerization of CA and monitor it *in situ* using nonresonant inelastic X-ray scattering spectroscopy (NRIXS). The time-evolution of the carbon core-electron excitation spectra shows the effects of two X-ray induced reactions: dimerization on a short time-scale and disintegration on a long time-scale. We used spectrum simulations of CA and its dimerization product, α-truxillic acid (TA), to gain insight into the dimerization effects. From the time-resolved spectra, we extracted component spectra and time-dependent weights corresponding to CA and TA. The results suggest that the X-ray induced dimerization proceeds homogeneously in contrast to the dimerization induced by ultraviolet light. We also utilized the ability of NRIXS for direct tomography with chemical-bond contrast to image the spatial progress of the reactions in the sample crystal. Our work paves the way for other time-resolved studies on chemical reactions using inelastic X-ray scattering.

Light-induced solid-state topochemical reactions are increasingly important in chemistry, since they hold promise for solvent-free green synthesis of materials^1^. Their products and reaction pathways are governed by the relative positions and orientations of the reacting molecules, i.e., by their crystal structure^2^,^3^,^4^,^5^. Typically, such reactions are induced via ultraviolet (UV) light through valence-electron excited states. A canonical example of such a UV-induced photochemical reaction is the dimerization of cinnamic acid^6^,^7^. However, the variations of the reaction pathways with varying photon energies are poorly understood, especially in the energy range of X-ray photons where they generally create mostly core-excited states. This is unfortunate, since the interaction of a material with these relatively high energy photons can lead to unexpected reactions^8^ and thus may serve to tailor novel reaction products.

Dimerization reactions, like all chemical reactions, are accompanied or driven by changes in the electronic structure. The electronic structure is commonly studied by X-ray absorption and electron energy loss spectroscopies (XAS and EELS, respectively), which are well established techniques for probing, for example, the oxidation and hybridization (e.g., sp^2^ vs. sp^3^) states of the elements present in a sample and are sensitive to very small sample densities and concentrations. XAS-based time-resolved techniques have developed into important tools for *in situ* studies of chemical reactions and catalytic processes^9^. However, when the interest is in organic compounds consisting of light elements, studies using these techniques are complicated due to the small penetration depth, which restricts sample environments and hinders bulk studies. Despite recent developments in this area^10^,^11^, there is a need for a complementary bulk-sensitive technique that is applicable in variable sample environments. This is exactly the role that nonresonant inelastic X-ray scattering (NRIXS) spectroscopy can fill.

NRIXS is a versatile technique for probing excitations of various energy ranges, from vibrational to valence- or core-level electronic excitations^12^,^13^,^14^. The information obtainable by measuring core-electron excitations with NRIXS (also referred to as X-ray Raman scattering, XRS) is similar to that by XAS and EELS. However, NRIXS has the major advantage that it can readily provide this information over macroscopic length scales and from bulk samples consisting of light elements, such as carbon, nitrogen, and oxygen, which constitute the overwhelming majority of interesting chemical substances. Thus, in a time-resolved NRIXS experiment, it is possible to observe the breaking of existing bonds or the formation of new bonds in organic compounds *in situ*, i.e., to follow typical chemical reactions with elemental sensitivity in real time. Furthermore, as shown recently^15^, NRIXS can be used to direct tomography giving data also with a spatial resolution, thus enabling imaging based on chemical-bond contrast. The ability to perform such combined time- and spatially-resolved experiments is tremendously valuable, but which has not been previously exploited.

The photodimerisation of cinnamic acid (C_9_H_8_O_2_) to truxillic acid is known to occur via [2 + 2]-cycloaddition induced by UV light illumination^6^,^7^. The reaction is depicted in Fig. 1, which shows how the aliphatic double bonds in the α-trans-cinnamic acid molecules form a cyclobutane ring in the product molecule, α-truxillic acid. Because the reaction involves 2 + 2 = 4 π-electrons, orbital symmetry precludes a thermal reaction pathway, whereas dimerization is induced easily by UV photons. Thus, the reaction proceeds through an excited state, and the potential-energy barriers of the ground state are overcome. The dimerization follows the topochemical principle of minimum atomic and molecular movement^5^, as the double bonds involved in the reacting monomers are close to each other and parallel. The reaction kinetics of this transformation can be described by a Johnson-Mehl-Avrami-Kolmogorov (JMAK) equation, as has been shown by X-ray diffraction^16^, nuclear magnetic resonance^17^, and Raman spectroscopy^18^.

In this article, we report our novel time-resolved inelastic X-ray scattering study of a solid-state topochemical reaction: The X-ray induced dimerization of α-trans-cinnamic acid (CA) to α-truxillic acid (TA). For the first time, this chemical reaction is both induced by X-rays and monitored *in situ* using their inelastic scattering. The dimerization is a surprising observation, since X-rays interacting with organic molecules have generally the tendency of dissociating the molecular structure instead of creating more complex ones. In addition, to follow the spatial progress of the dimerization, we have utilized the ability of NRIXS to direct tomography with a chemical-bond contrast^15^. Thus, as a time-resolved imaging experiment on a chemical reaction, this study is also an important step towards the realization of the full potential of NRIXS. The sub-minute time-resolution of this experiment is readily improvable to a second-scale, and the spatial-resolution of ~50 µm is foreseen to reach 1–10 µm. First, we present the evolution of the carbon core-electron excitation spectrum from the CA sample as a function of time and discuss the observed dimerization effect in the spectrum with the help of simulations and reference TA spectra. Next, we present the conversion curves of the reactants and the images of the CA crystal showing the formation of TA and the products of a subsequent disintegration reaction. Finally, we discuss the reaction kinetics and their differences compared to traditional UV-light induced dimerization.

## Results

### X-ray-induced dimerization

The time-evolution of the carbon core-electron excitation spectra measured from the crystalline samples of α-trans-cinnamic and α-truxillic acid are displayed in Fig. 2 panels (a) and (b), respectively. These spectra are from here on referred to as the CA and TA spectra. The beginning of the X-ray irradiation of the samples corresponds to the origin of the time-scales (0–196 min and 0–375 min). The spectra are normalized to unit area in the energy range 282–296 eV.

In the CA spectrum, during the first ~17 min of irradiation, the maximum of the lowest-energy peak (the peak labelled A) shifts from 284.8 eV to 285.2 eV and the intensity of the peak decreases. This is in contrast to the TA spectrum, in which the maximum of this peak is at 285.2 eV from the beginning, and the intensity does not change much during this time. The second-lowest energy peak (labelled B) of the spectra behaves in a similar fashion: In CA, its maximum shifts from 288.2 eV to 288.6 eV, whereas in TA, its maximum remains at 288.6 eV. In both the CA and TA spectra, there is also a decrease in the intensity of this peak that is more pronounced in TA. A third notable change in the CA spectrum occurs at 291.0 eV, where a prominent new peak emerges (labelled C). In the TA spectrum this peak is present from the beginning of the measurement. Additionally, in the CA spectrum, the overall intensity in the range 290–296 eV increases. Thus, after ~17 min of X-ray irradiation, the CA spectrum has changed to resemble the TA spectrum at *t* = 0 min. To illustrate this result, the CA spectrum at 17 min and the TA spectrum at 0 min are shown in panel (c) of Fig. 2. The mutual resemblance of the spectra indicates that the X-ray irradiation induces the dimerization of CA and produces TA. However, full conversion is not reached, for reasons explained below. The mismatch of the intensities of peak B remains unexplained.

After a longer irradiation time, the CA spectrum evolves further, albeit at a slower rate. This indicates that the overall X-ray induced reaction produces final products (FP) other than α-truxillic acid. The overall reaction most probably includes both CA → TA → FP and CA → FP paths. The changes in the CA spectrum are practically identical to those in the TA spectrum, see the panel (c) of Fig. 2 for a comparison of the spectra from the CA sample at *t* = 196 min and from the TA sample at *t* = 212 min. The main changes are a decrease of peaks A and B, and the emergence of a pronounced new peak (labelled D), which suggests a disintegration reaction. The energy of this new peak (287.5 eV) coincides with that of the main peak of the spectrum of carbon monoxide^19^,^20^ (Supplementary Fig. 1), suggesting carbon monoxide as a plausible final product.

To better understand the spectral changes caused by the dimerization, we performed simulations for the spectra of CA and TA. The results of these simulations in the energy range 283–291 eV are shown in Fig. 3 panel (a) with a comparison to the measured CA and TA component spectra (see the next section for component extraction). The simulations reproduce accurately the experimentally observed shift of the maximum of peak A to higher energy in the course of the dimerization, but not the intensity decrease. For this reason, the interpretations outlined below remain somewhat tentative. The mismatch in the intensity behaviour might occur, because in the simulation, only the dipole excitations are accounted for, whereas in the experiment, also nondipole excitations are present due to significantly nonzero momentum transfer. The normalizations of the spectra are not comparable, as the simulations do not perform accurately with higher-energy excitations, which is also the reason for showing the spectra only up to 291 eV. Slight differences between the experiment and the simulation are also expected because the simulation is performed with optimized geometries of isolated molecules, whereas in the experiment, the samples are in the crystalline form.

**Figure 1:**
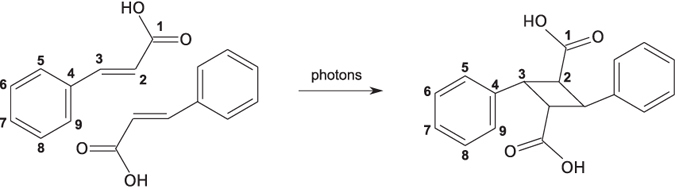
Photodimerization of α-trans-cinnamic acid to α-truxillic acid. The numbers refer to the carbon atoms, for which individual excitation spectra are shown in Fig. 3.

Panels (b–d) of Fig. 3 show the spectral contributions from individual carbon atoms from the simulation. Distinguishing these contributions is possible because the core-hole is localized, and its location can be chosen in the simulation to be at a given atom. Note that it is sufficient to consider only the atoms in α-truxillic acid that are labelled in Fig. 1, because the molecule is centrosymmetric. A qualitative comparison of the individual spectral contributions reveals that the observed effect on the spectra associated with the dimerization is mainly caused by the contributions from carbon atoms 1–3 and, to a somewhat lesser extent, by atoms 4, 7, and 9 (in the benzene ring).

The observed energy shift of the lowest-energy peak A maximum (284.8 eV → 285.2 eV) reflects the massive modifications of the electronic structure of carbon atoms 2 and 3 caused by the dimerization; the orbital hybridization of both atoms changes drastically from sp^2^ to sp^3^. The aliphatic carbon-carbon double bonds of CA transform into carbon-carbon single bonds of the central cyclobutane moiety of TA. Interestingly, the spectra of the aromatic carbon atoms 4, 7, and 9 also undergo considerable modifications upon dimerization, whereas the changes for atoms 5 and 6 are far less pronounced, and for atom 8, there are practically no changes at all. The shift of peak B (288.2 eV → 288.6 eV) is associated with the changes of the electronic structure of the carbonyl carbon atom 1; this may appear puzzling as the carboxyl groups do not undergo any chemical modifications.

All of these observations can be rationalized readily when taking into account that CA is an example of a conjugated π-electron system involving all carbon atoms. Following this line of reasoning, one might be tempted to predict a relative sensitivity of aromatic carbon atom 4 as well as of the carbon atoms in the ortho and para positions (5/9 and 7, respectively) to alterations of the p-orbital interactions as a consequence of mesomeric effects, while the carbon atoms at the meta positions 6/8 should only be affected to a small extent, as is actually the case. The electronic structure of the TA molecule is entirely different because the cyclobutane ring effectively precludes p–p interactions along the long molecular axis and between the aromatic substituents and the carboxyl groups. For recent simulation results of the electronic structure of CA, see ref. 21.

### Component spectra and conversion curves

To further analyse the CA and TA time-resolved spectra series, we used nonnegative matrix factorization (NMF), see e.g., ref. 22. This method allowed us to obtain spectra from the reactants and products and their time-dependent weights, even if the compounds did not exist in the sample in the pure form at any instant. Unlike the principal component analysis (PCA) widely used for this kind of task, NMF provides a straightforward spectrum decomposition, whose results can be directly interpreted as the component spectra and their weights. The differences of NMF and PCA for spectral decomposition were studied in ref. 23. See the Supplementary Information for details of NMF applied to our case.

**Figure 2:**
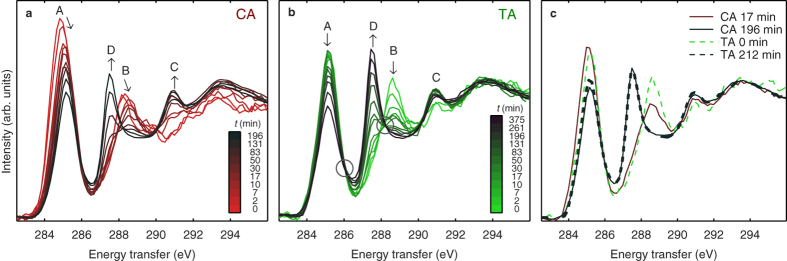
The time-resolved carbon core-electron excitation spectra from the experiment. (**a,b**) The spectra of the CA and TA samples, respectively, after the X-ray irradiation times as shown. The isosbestic points in the TA spectra series are marked with circles. (**c**) Comparisons of the spectra of the CA and TA samples at selected times.

**Figure 3:**
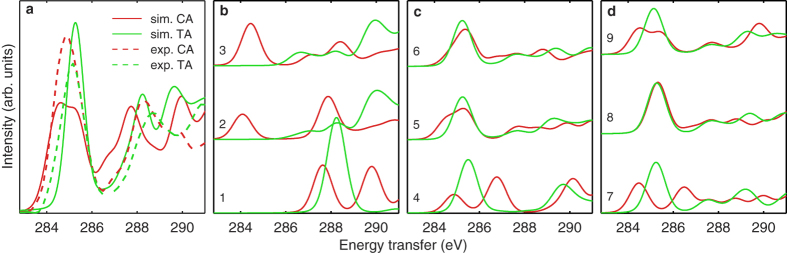
The spectra of CA and TA from the simulations. (**a**) Comparison of the spectra from the simulations (solid lines, the average over all carbon atoms) to the spectra from experiment (dashed lines, the components extracted from the time-resolved spectra of the CA sample). (**b**–**d**) The individual spectral contributions from each carbon atom of CA and TA from simulations. The numbering refers to the atom labels in Fig. 1.

As shown in Fig. 2, in the TA time-resolved spectra series there are two isosbestic points at 286.1 eV and 288.2 eV, whereas in the CA spectra there are no isosbestic points. This implies that the TA spectra series contains two components, whereas the CA spectra series contains at least three, which is in line with the reactions TA → FP and CA → TA → FP, respectively. Therefore, we applied NMF to the CA spectra series to extract three component spectra and their time-dependent weights and to the TA spectra series to extract two of them. We also utilized the bootstrap method in the NMF extraction to take into account the statistical uncertainty of the spectra, thus obtaining error estimates for the component spectra and weights, but found them to be negligible. We note that other error sources are not accounted for, such as the uncertainty in the time-points (due to finite time for energy scans) or dose rate variation in the sample (due to the attenuation of X-rays and variations in their incident intensities).

**Figure 4:**
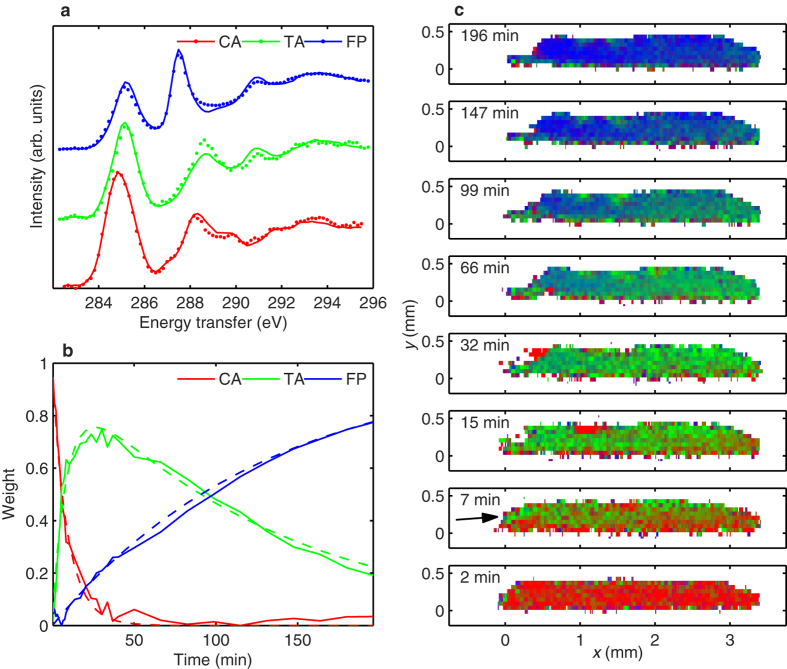
Results of the NMF analysis and spectral imaging of the CA sample. (**a**) The extracted component spectra (solid lines) compared to individual spectra of selected times (points). The spectra are offset for clarity. (**b**) The component weights as a function of time: extracted using NMF (solid lines), and by a fitted model (dashed lines). (**c**) Imaging of the progression of the chemical composition in the CA crystal. Red, green, and blue channel correspond to the weights of CA, TA, and FP component spectrum, respectively. The X-ray beam enters the crystal from left.

The component spectra of CA, TA, and FP and their weights, extracted from the CA time-resolved spectra series, are shown in panels (a) and (b) of Fig. 4, respectively. In panel (a), there are also selected individual spectra from the spectra series shown with points (CA from cinnamic acid sample at time 0 min, TA and FP from truxillic acid sample at times 0 min and 375 min, respectively). The weights illustrate that the dimerization, i.e., the CA → TA reaction, occurs rapidly compared to the TA → FP reaction: The weight of 0.5 is reached by TA in ~7 min, whereas by FP it takes ~75 min to reach the same weight. We note that these curves are the weights of the component spectra (normalized to unity for each time point) as a function of time, but expect them to correspond closely to the fractions of the compounds and conversion curves. The components and weights from the TA time-resolved spectra are presented in Supplementary Fig. 2.

### Kinetics of the reactions

To quantify the reaction kinetics, we fit a reaction model to the conversion curves. The UV light-induced dimerization of α-trans-cinnamic acid has been previously shown to follow JMAK kinetics^16^,^17^, in which the reaction proceeds via nuclei formation and their subsequent growth, and the TA fraction obeys the relation


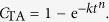


Here, *k* is the growth rate constant, *t* the time from the beginning of the reaction, and *n* the Avrami exponent, which can be related to the dimensionality of the growth of the nuclei. In our case, as the competing CA → FP and TA → FP disintegration reactions are also present, we model the reactions in the CA sample using differential equations as follows:


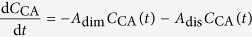



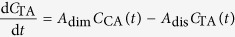



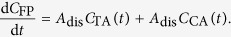


To include the JMAK kinetics for the dimerization we set *A*_dim_ = *knt*^*n*−1^. In the equations *C*_CA_, *C*_TA_, and *C*_FP_ are the weights/fractions of CA, TA, and FP, respectively, and *A*_dis_ is the reaction rate constant for disintegration (the same for both CA → FP and TA → FP). By fitting the numerical solutions of these equations to the conversion curves from the experiment, we obtain the Avrami exponent *n* = 0.93. See Supplementary Table 1 for all parameters of the fits. The modelled conversion curves using the parameters from the fit are shown in Fig. 4(b) with dashed lines and agree well with the curves from the experiment. Similar modelling for the disintegration in the TA sample was performed, see Supplementary Fig. 2.

### Imaging with chemical-bond contrast

To examine the progress of the reactions in the cinnamic acid crystal in spatial dimensions we utilized the imaging ability of NRIXS^15^. In this direct tomography technique the contrast originates from the chemical bonding information contained in a hyperspectral image, which is obtained using a two-dimensional detector recording the scattered X-rays. Thus, the image is a projection of the X-ray illuminated volume of a sample.

In the present case this technique allowed the mapping of a section of the sample crystal based on the CA, TA, and FP component weights. The same dataset used for the above kinetics analysis was analysed retaining it in the hyperspectral image form, i.e., having a separate spectrum in each pixel (and for each time-point). These hyperspectral images were then fitted with the component spectra of CA, TA, and FP from the above NMF analysis, giving the component weights in each pixel, which are shown in Fig. 4(c) represented with colors.

## Discussion

Previous studies^16^,^17^ on the kinetics of the UV light induced α-trans-cinnamic acid dimerization have resulted in Avrami exponents *n* = 1.43 ± 0.08 and *n* = 1.66 ± 0.10. These kinds of values, between one and two, can originate from a hybrid reaction mechanism^16^, a combination of both homogeneous reaction (case *n* = 1) and one-dimensional growth from continuously forming nuclei (case *n* = 2). The value of the Avrami exponent near one in our experiment suggests that the reaction may proceed more homogeneously with the rate being linearly dependent on the reactant concentration.

The difference between the present and previous results is most likely due to different reaction mechanisms for X-ray and UV irradiation. The UV initiated process contrasts the X-ray case in that the initial step can only be a valence ionization or excitation, and that no high-energy photo-electrons are produced. Although the specific pathways of the X-ray induced dimerization were not studied in this work, it is evident that the first step in them is either photoelectric absorption or inelastic scattering, as these are the only interactions that deposit energy to the sample. In the case of a 10-keV photon interacting with carbon or oxygen atom the dominant process is absorption^24^, in which a singly-ionized state of the sample molecule is produced together with a photo-electron of high energy (nearly the incident 10 keV). The ionization occurs most probably from 1s-orbital, and the resulting core-excited states decay in turn dominantly via the Auger process, in which an Auger-electron is emitted and the molecule is left in a doubly ionized state. In the case of a 10-keV photon and a hydrogen atom, the dominant interaction is inelastic scattering yielding a scattered photon with high energy and an ejected Compton-electron. All of the released high-energy photo-electrons cause chain-reactions of sequential ionizations and excitations as well as bremsstrahlung in the sample^24^. Some of these secondary excitations and ionizations can be the same valence excitations caused by UV illumination, which is thus the simplest explanation for the fact that dimerization is induced with both X-ray and UV irradiation. Naturally, the free radicals that are possibly produced from the disintegration of the ionised or excited states can be very reactive breaking bonds and affecting the reaction. The lower value n = 0.93 for Avrami exponent found in the X-ray experiment, compared to the UV experiments, might be explained by noting the high penetration depth of X-rays and also that the valence excitations are dominantly created as a secondary process, initiating a more homogenous dimerization of the sample.

Cinnamic acid dimerization kinetics has not reported to depend on the dose rate. However, for future research on the reaction path, a more complete study on the dependence of the kinetics on the wavelength of the inducing radiation is desirable, along on the dependence on the dose rate and sample temperature. For example, irradiation with soft X-rays does not produce high-energy photo-electrons to create subsequent valence excitations, and neither they can be created directly by inelastic scattering. Thus, a similar experiment to the present one performed with X-ray absorption spectroscopy, i.e., with incident energy around 280 eV, could provide more information on the dimerization mechanism.

The imaged section of the cinnamic acid crystal in Fig. 4(c) shows how the formation of TA and FP occurs slightly faster in the left side than on the right side of the crystal, as the X-ray beam enters from left and dose rate is higher on that side. As a remark, at 7 min a small part on the left edge (indicated with an arrow) shows high conversion to TA, and in 15–32 min this part seems to break away from the crystal. The studies of UV induced dimerization have reported a fragmentation of crystals to pieces of low- and high-conversion due to mismatch of the CA and TA crystal lattices^16^, which seems a plausible explanation for the observed break off.

In conclusion, we have shown that α-trans-cinnamic acid undergoes an X-ray-induced dimerization reaction using *in situ* inelastic X-ray scattering spectroscopy. The time-resolved carbon core-electron excitations spectra show that the X-rays induce a rapid dimerization reaction and a slower disintegration reaction. The effect of the dimerization on the spectrum was studied with the help of simulations. The kinetics of the reactions was investigated by extracting the time-dependent weights of the α-trans-cinnamic acid, α-truxillic acid, and final products from the time-resolved spectra. Furthermore, we have shown that NRIXS is capable to time-resolved imaging of the breakup and formation of intermolecular bonds, i.e., chemical reactions. Our work paves the way to other novel time-resolved *in situ* studies of X-ray-induced chemical reactions using NRIXS.

## Methods

### Sample preparation

Crystals of α-trans-cinnamic acid (CA) were grown from acetone solution of cinnamic acid by slowly evaporating the solvent, typically resulting in crystals of size 5 × 5 × 0.5 mm^3^. α-truxillic acid (TA) crystals were obtained from CA crystals by illumination with UV light from a xenon arc lamp for ~9 h. The Raman and IR spectra of CA and TA were found to be in accordance with the literature^25^.

### Experiment

NRIXS is performed with synchrotron radiation, and it has become feasible at 3rd generation synchrotron radiation facilities and continually more popular owing to developments in the instrumentation^26^,^27^,^28^,^29^. In NRIXS, an X-ray photon scatters inelastically from the sample system and transfers only part of its energy to an excitation of the system. Thus, the energy of the incident X-rays can be chosen independent of the excitation energy range of interest. Using incident X-rays of ~10 keV, NRIXS has a probing depth in the mm-range for solid or liquid organic samples. Recently, NRIXS has been used, for example, in studies of water and carbon dioxide in extreme conditions^30^,^31^, of gas-phase samples^32^,^33^, and in a novel imaging technique, where the contrast originates from the chemical bonding^15^.

The NRIXS measurements of this work were performed at the beamline ID20 of the European Synchrotron Radiation Facility. The sample crystals were mounted on the cold finger of a He flow cryostat, which was cooled to ~10 K to slow down the irradiation induced reactions. The series of time-resolved spectra were obtained by 29 consecutive energy scans for CA and by 40 scans for TA. The time-resolution of the spectra series was set by the the scan time, which was varied: for the first 10 scans it was 53 s, for the second 10 scans 199 s, and for the rest 975 s. In this experiment the scan time of 53 s was needed to acquire a sufficient number of counts to the spectra, but by optimizing the measurement the time-resolution could be readily improved to a second-scale at the same beamline. The average dose rate by X-rays on the samples was estimated to be 20 kGys^−1^, giving total doses of 0.2 GGy for CA and 0.4 GGy for TA during the measurements.

The incident X-ray beam was generated with four undulators, monochromatized with a Si(111) premonochromator and a Si(311) channel-cut monochromator, and focused on the sample to a size of 150 × 800 µm^2^ (vertical × horizontal full width at half maximum, FWHM). The spectra were measured by scanning the energy of the incident beam at such energies above the pass energy of the analyser crystals of the spectrometer (9.69 keV) that the desired energy loss range (282–298 eV) was obtained. The angular positions of the 12 Si(660) analyser crystals set the momentum transfer to 6.1 ± 0.6 Å^−1^ (s.d.). An average energy resolution of 0.7 eV (FWHM) was determined from the elastic lines.

Data analysis was performed with the software package XRSTools^34^ and MATLAB and Statistics Toolbox Release 2014a. The imaging of the sample was performed in the 2D sectioning mode described in ref. 15, and the analysis for imaging followed the super-resolution method as in ref. 34. A perspective correction of the low-resolution images (due to different angles of view of the analyser crystals) was performed by applying an affine transformation on them.

### Simulations

The spectrum simulations were performed on optimized geometries of single CA and TA molecules utilizing the transition potential half-hole approximation using Gaussian and augmented plane wave method^35^. The CP2K code package^36^,^37^ and the Perdew–Burke–Ernzerhof (PBE) exchange-correlation functional^38^ were used in the dipole calculations, for each carbon atom separately. The augmented correlation-consistent polarized valence quintuple-*ξ* (aug-cc-pV5Z) basis sets by Dunning *et al.*^39^ were used for the absorbing carbon, whereas polarized triple-*ξ* basis sets optimized for condensed matter (TZV2P-MOLOPT)^40^ and GTH pseudopotentials^41^ were used for the other atoms. The resulting delta peak spectra were convoluted with a Lorentzian line shape with a FWHM of 0.12 eV and with a Gaussian with a FWHM of 0.9 eV to account for the life-time, instrumental, and vibrational broadenings and shifted by −1.1 eV to align them with the measured spectra.

## Additional Information

**How to cite this article**: Inkinen, J. *et al.* X-ray induced dimerization of cinnamic acid: Time-resolved inelastic X-ray scattering study. *Sci. Rep.* **5**, 15851; doi: 10.1038/srep15851 (2015).

## Supplementary Material

Supplementary Information
