# Supplementary material for: X-ray induced dimerization of cinnamic acid: Time-resolved inelastic X-ray scattering study
Source: Sci Rep. 2015 Nov 16;5:15851. doi: 10.1038/srep15851 (PMC4644965; doi:10.1038/srep15851)
Supplement: Supplementary Information [file srep15851-s1.pdf]

# X-ray induced dimerization of cinnamic acid: Time-resolved inelastic X-ray scattering study
# Supplementary Information

Juho Inkinen,[1,*] Johannes Niskanen,[1] Tuomas Talka,[1] Christoph J. Sahle,[2] Harald Müller,[2] Leonid Khriachtchev,[3] Javad Hashemi,[1] Ali Akbari,[1] Mikko Hakala,[1] and Simo Huotari[1]

[1]*Department of Physics, P.O. Box 64, FI-00014 University of Helsinki, Helsinki, Finland*
[2]*ESRF - The European Synchrotron, CS 40220, F-38043 Grenoble Cedex 9, France*
[3]*Department of Chemistry, P.O. Box 55, FI-00014 University of Helsinki, Helsinki, Finland*
(Dated: August 7, 2015)

## FINAL PRODUCT IDENTIFICATION

To identify the final products of the disintegration, we show in Fig. 1 our NRIXS spectra from α-truxillic acid and the final products (extracted with nonnegative matrix factorization) and reference spectra from Refs. [1–3]. The reference spectra have been measured with electron-energy-loss spectroscopy from gas-phase samples, and were obtained from an online database [4]. The reference spectra have been measured near dipole limit, whereas our spectra are measured with (significantly) nonzero momentum transfer, but this may cause differences only in line intensities. We note also the solid-state effects may influence our spectra but not the reference spectra. Nevertheless, although the reference spectra are not to straightaway comparable to our spectrum, for identification purposes they are sufficient.

As seen in the Fig. 1, the prominent peak emerged during X-ray irradiation at 287.5 eV coincides with the main peak of the reference carbon monoxide (CO) spectrum, suggesting that CO is a final product of the disintegration reaction. We emphasize that the actual disintegration reaction may well be comprised of several steps in possibly several pathways, in which radicals and various intermediates may be present, but in which lasting CO is eventually produced. This is the case in the ionization-induced fragmentation of gas-phase cinnamic acid [5], where two pathways are present, producing H, OH, CO, $CO_2$, $C_2H_2$, $C_4H_3^+$, $C_7H_7^+$, and $C_8H_6^+$. However, in our experiment in solid-state, acetylene or carbon dioxide ($C_2H_2$ or $CO_2$, respectively) seem not be (long-living) final products, as our spectra do not show any intensity gain at the energies where the corresponding reference spectra have prominent peaks. Actually, our spectra show intensity increase exclusively at 287.5 eV, corresponding to CO, suggesting it to be the only carbon containing compound that is eventually produced in significant amounts.

Intensity decrease, in turn, occurs at two regions, i.e. on the main peak at 284–286 eV and at 288.2–290.0 eV. As can be seen from the simulation results in Fig. 3, the first of these regions gains its intensity from the carbon atoms in the phenyl rings (atoms 4–9), and the second from the carbons in the aliphatic part (atoms 1–3). This suggests that both these parts of the TA molecule seem to be sensitive to disintegration, neither of them being clearly more resistant than the other. Despite the intensity decrease at these regions (and the emerged CO peak), the FP spectrum still resembles the TA spectrum. This might be explained, as it is not stoichiometrically possible for truxillic acid to convert to carbon monoxide, but there are excess carbon atoms, which may, on average, remain the truxillic acid molecular structure.

## NONNEGATIVE MATRIX FACTORIZATION

In nonnegative matrix factorization (NMF) a nonnegative dataset matrix $X$ is approximately factorized into two nonnegative matrices $W$ and $H$, i.e. $X \approx WH$. In the case of a time-resolved series of spectra, $W$ can be taken to represent component spectra and $H$ their time-dependent weights. When $X$ has dimensions $n \times m$ ($n$ points in energy and $m$ points in time) and the number of components is $k$, the dimensions of $W$ and $H$ are $n \times k$ and $k \times m$, respectively. Recently NMF has been used to analyze various kinds of datasets, e.g., pH- and concentration-varied spectra series [6–10], and time-resolved spectra series [11]. See also e.g. Ref. 12 for similar methods.

The NMF was performed using its implementation in MATLAB and Statistics Toolbox Release 2012a with alternating least-squares algorithm. When factorizing the TA series, its first and last spectra were used to form initial guess for $W$. In the case of CA series, its first spectrum and the spectra components extracted from the TA series were used. The obtained spectra components were normalized to unit area, and the sum of the component weights to unity at each timepoint. For reference we utilized also a modification of NMF from Ref. 13, so called versatile

sparse matrix factorization, that allows relaxing the nonnegativity constraints and adding sparsity and smoothness constraints on $W$ and $H$. Similar results were obtained with this method to those with NMF, but without the need of using initial guesses for the components, the results deviating mostly only on the TA component weights.

Fig. 2 shows the components and their weights extracted with NMF from the truxillic acid series. In panel (a) there are also selected individual spectra from the spectra series shown with points (TA and FP at times 0 min and 375 min, respectively)

## REACTION KINETICS IN TRUXILLIC ACID

The disintegration reaction in the initial α-truxillic acid sample is modeled with

$$\frac{\mathrm{d}C_{\mathrm{TA}}}{\mathrm{d}t} = -A_{\mathrm{dis}} C_{\mathrm{TA}}(t)$$
$$\frac{\mathrm{d}C_{\mathrm{FP}}}{\mathrm{d}t} = A_{\mathrm{dis}} C_{\mathrm{TA}}(t),$$

The modeled conversion curves following the parameters of the fit are plotted in Fig. 2 (b) with dashed lines. Table I presents all parameters of the fits.

| | | Present study | | | Previous studies | |
|---|---|---|---|---|---|---|
| | | $k$ ($10^{-3}$ $s^{-n}$) | $n$ | $A_{\mathrm{dis}}$ ($10^{-4}$ $s^{-1}$) | $n^a$ | $n^b$ |
| CA sample | CA→TA | 2.66 | 0.90 | - | $1.43 \pm 0.08$ | $1.66 \pm 0.10$ |
| | CA/TA→FP | - | - | 1.6 | - | - |
| TA sample | TA→FP | - | - | 1.6 | - | - |

[a] Reference [14]
[b] Reference [15]

TABLE I. Parameters of kinetics model obtained by fitting to the experimental conversion curves.

* juho.inkinen@helsinki.fi

[1] A. Hitchcock and C. Brion, J. Electron. Spectrosc. Relat. Phenom. **18**, 1 (1980).
[2] A. Hitchcock and C. Brion, J. Electron. Spectrosc. Relat. Phenom. **10**, 317 (1977).

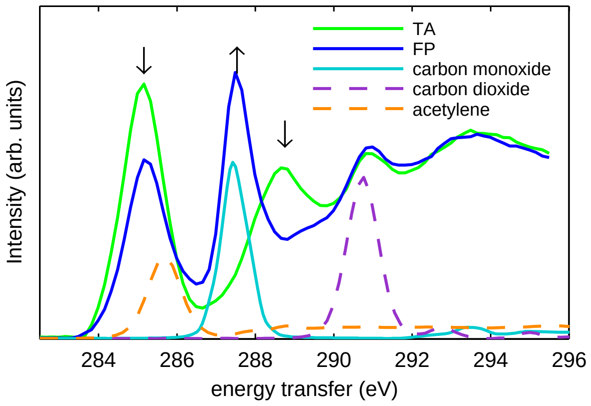


FIG. 1. The spectra of α-truxillic acid and final products from the present experiment (TA and FP, respectively) compared to reference spectra. The spectra are normalized to unit area on the shown region, after which the reference spectra are divided by a factor of 10 for clarity. The arrows indicate changes on TA→FP.

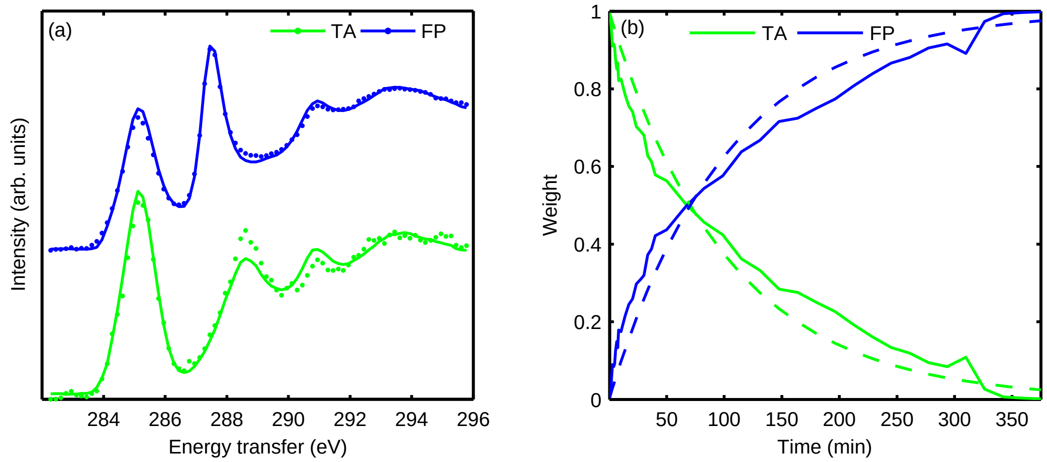


FIG. 2. Panel (a): The component spectra extracted with NMF from the truxillic acid time-resolved spectra series (solid lines) and selected individual spectra (points). Panel (b): The component weights (conversion curves) as a function of time from truxillic acid spectra series: extracted with NMF (solid lines), and by fitted model (dashed lines).

[3] R. McLaren, S. A. C. Clark, I. Ishii, and A. P. Hitchcock, Phys. Rev. A **36**, 1683 (1987).
[4] A. Hitchcock and D. Mancini, “Gas Phase Core Excitation Database,” (1980), http://unicorn.mcmaster.ca/corex/cedb-title.html.
[5] B. Schaldach and H.-F. Grützmacher, Org. Mass Spectrom. **15**, 175 (1980).
[6] S. K. Srivastava, S. Niebling, B. Küstner, P. R. Wich, C. Schmuck, and S. Schlücker, Phys. Chem. Chem. Phys. **10**, 6770 (2008).
[7] S. Niebling, S. K. Srivastava, C. Herrmann, P. R. Wich, C. Schmuck, and S. Schlucker, Chem. Commun. **46**, 2133 (2010).
[8] S. Niebling, H. Y. Kuchelmeister, C. Schmuck, and S. Schlucker, Chem. Commun. **47**, 568 (2011).
[9] W. Xie, C. Herrmann, K. Kömpe, M. Haase, and S. Schlücker, J. Am. Chem. Soc. **133**, 19302 (2011), pMID: 22053855, http://dx.doi.org/10.1021/ja208298q.
[10] I. Kopriva, I. Jerić, and A. Cichocki, Chemometr. Intell. Lab. **97**, 170 (2009).
[11] P. Liu, X. Zhou, Y. Li, M. Li, D. Yu, and J. Liu, Anal. Methods **5**, 4454 (2013).
[12] A. de Juan and R. Tauler, Crit. Rev. Anal. Chem. **36**, 163 (2006), http://dx.doi.org/10.1080/10408340600970005.
[13] Y. Li and A. Ngom, Source code for biology and medicine **8**, 1 (2013).
[14] J. B. Benedict and P. Coppens, J. Phys. Chem. A **113**, 3116 (2009), http://dx.doi.org/10.1021/jp809170t.
[15] M. Bertmer, R. C. Nieuwendaal, A. B. Barnes, and S. E. Hayes, J. Phys. Chem. B **110**, 6270 (2006), pMID: 16553444, http://dx.doi.org/10.1021/jp057417h.
